# Supplementary material for: Intervention Effect of Non-Invasive Brain Stimulation on Cognitive Functions among People with Traumatic Brain Injury: A Systematic Review and Meta-Analysis
Source: Brain Sci. 2021 Jun 24;11(7):840. doi: 10.3390/brainsci11070840 (PMC8301762; doi:10.3390/brainsci11070840)
Supplement: Supplementary file 1 [file brainsci-11-00840-s001.zip › brainsci-1234358-supplementary.pdf]

Supplementary Materials

Sensitivity Analyses

First, Motes, *et al.* [1] was deleted from the initial nine studies as the study's design was prospective, single-blinded study (non-randomised control trial). The meta-analysis on the eight studies revealed a significant positive and small effect of active NIBS on overall cognitive outcomes ($k = 8$, $g = 0.292$, 95% CI 0.033 to 0.550, $p = 0.027$; see Figure S1).

Study name	Statistics for each study			
	Hedges's g	Lower limit	Upper limit	p-Value
Lesniak et al. 2014	0.272	-0.409	0.953	0.434
Ulam et al. 2015	0.415	-0.224	1.053	0.203
Leung et al. 2016	0.659	-0.163	1.481	0.116
Lee and Kim 2018	1.150	0.034	2.266	0.043
Hoy et al. 2019	0.294	-0.415	1.004	0.416
Neville et al. 2019	0.169	-0.450	0.789	0.592
Rao et al. 2019	-0.107	-0.738	0.524	0.739
Siddiqi et al. 2019	0.102	-0.932	1.136	0.847
	0.292	0.033	0.550	0.027

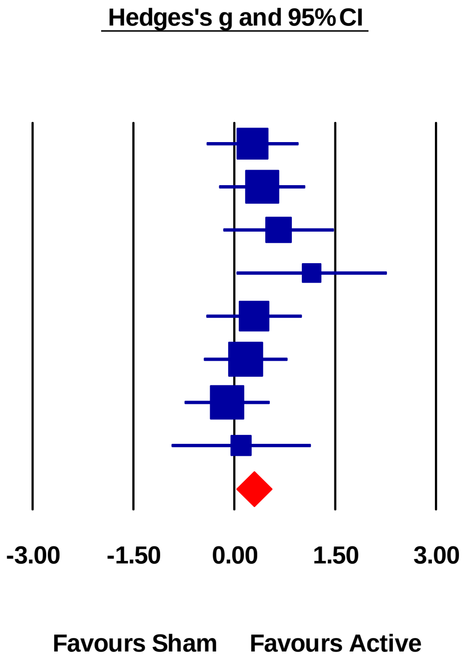

Random effects analysis Tau=0

Figure S1. The Effect of Non-Invasive Brain Stimulation (NIBS) on overall Cognitive Outcomes: A Forest Plot.

Second, two studies (Motes, *et al.* and Leung, *et al.*) were removed from the analysis as they had high risk of bias or could not be assessed (not applicable) [1,2]. The meta-analysis on the seven studies revealed a non-significant effect of active NIBS on overall cognitive outcomes ($k = 7$, $g = 0.251$, 95% CI -0.021 to 0.524, $p = 0.071$; see Figure S2).

Study name	Statistics for each study			
	Hedges's g	Lower limit	Upper limit	p-Value
Lesniak et al. 2014	0.272	-0.409	0.953	0.434
Ulam et al. 2015	0.415	-0.224	1.053	0.203
Leung et al. 2016	0.659	-0.163	1.481	0.116
Lee and Kim 2018	1.150	0.034	2.266	0.043
Hoy et al. 2019	0.294	-0.415	1.004	0.416
Neville et al. 2019	0.169	-0.450	0.789	0.592
Rao et al. 2019	-0.107	-0.738	0.524	0.739
Siddiqi et al. 2019	0.102	-0.932	1.136	0.847
	0.292	0.033	0.550	0.027

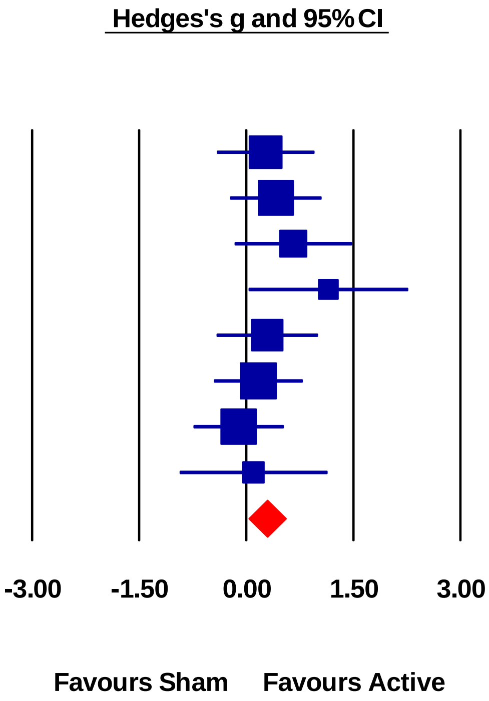

Random effects analysis Tau=0

Figure S2. The Effect of Non-Invasive Brain Stimulation (NIBS) on overall Cognitive Outcomes: A Forest Plot.

References

1. Motes, M.; Spence, J.; Yeatman, K.; Jones, P.; Lutrell, M.; O'Hair, R.; Shakal, S.; DeLaRosa, B.; To, W.; Vanneste, S.; et al. High-Definition Transcranial Direct Current Stimulation to Improve Verbal Retrieval Deficits in Chronic Traumatic Brain Injury. *J. Neurotrauma* **2020**, *37*, 170-177, doi:10.1089/neu.2018.6331.
2. Leung, A.; Shukla, S.; Fallah, A.; Song, D.; Lin, L.; Golshan, S.; Tsai, A.; Jak, A.; Polston, G.; Lee, R. Repetitive Transcranial Magnetic Stimulation in Managing Mild Traumatic Brain Injury-Related Headaches. *Neuromodulation: Technology at the Neural Interface* **2016**, *19*, 133-141, doi:10.1111/ner.12364.
